# Laser Welding of SLM-Manufactured Tubes Made of IN625 and IN718

**DOI:** 10.3390/ma12182967

**Published:** 2019-09-12

**Authors:** Torsten Jokisch, Angelina Marko, Sergej Gook, Ömer Üstündag, Andrey Gumenyuk, Michael Rethmeier

**Affiliations:** 1 Siemens AG, Huttenstr. 12, 10553 Berlin, Germany; torsten.jokisch@siemens.com; 2 Fraunhofer Institut für Produktionsanlagen und Konstruktionstechnik, Pascalstr. 8-9, 10587 Berlin, Germany; sergej.gook@ipk.fraunhofer.de (S.G.); oemer.uestuendag@ipk.fraunhofer.de (Ö.Ü.); michael.rethmeier@bam.de (M.R.); 3 Bundesanstalt für Materialforschung, Unter den Eichen 87, 12205 Berlin, Germany; andrey.gumenyuk@bam.de; 4 Füge-und Beschichtungstechnik, TU Berlin, Pascalstr. 8-9, 10587 Berlin, Germany

**Keywords:** additive manufacturing, welding, joining, powder bed fusion

## Abstract

The advantage of selective laser melting (SLM) is its high accuracy and geometrical flexibility. Because the maximum size of the components is limited by the process chamber, possibilities must be found to combine several parts manufactured by SLM. An application where this is necessary, is, for example, the components of gas turbines, such as burners or oil return pipes, and inserts, which can be joined by circumferential welds. However, only a few investigations to date have been carried out for the welding of components produced by SLM. The object of this paper is, therefore, to investigate the feasibility of laser beam welding for joining SLM tube connections made of nickel-based alloys. For this purpose, SLM-manufactured Inconel 625 and Inconel 718 tubes were welded with a Yb:YAG disk laser and subsequently examined for residual stresses and defects. The results showed that the welds had no significant influence on the residual stresses. A good weld quality could be achieved in the seam circumference. However, pores and pore nests were found in the final overlap area, which meant that no continuous good welding quality could be accomplished. Pore formation was presumably caused by capillary instabilities when the laser power was ramped out.

## 1. Introduction

The ongoing improvement in metal additive manufacturing (AM) processes allows a rapid and near-net-shape production of complex parts with good mechanical properties. When it comes to industrial applications, the selective laser melting (SLM) process is especially popular because its production of more detailed and accurate parts [1]. For example, machining tools with inner cooling channels can be made through SLM today [2]. The maximum part size is limited by the SLM machines’ building chamber dimensions. Therefore, possibilities to overcome these limitations need to be developed [3]. To combine several parts to a bigger structure through welding would be a feasible strategy to improve the maximum size of SLM components. A large amount of research effort is needed to develop strategies and standards for that endeavor, as only few investigations on the joining of SLM parts can be currently found in the literature [4].

## 2. State-of-the-Art

SLM produces complex structures through layer-by-layer deposition in a powder bed by means of a laser. After one powder layer is melted, a new powder layer on top of the structure is applied, and the building platform moves down as far as the last layer height makes it necessary. As the origin of production, a three-dimensional (3D) CAD model is used, which has to be sliced prior to the process in several layers with specific heights. To prevent oxidations of the molten powder, the process chamber is filled with shielding gas to protect the molten pool. In some cases, post-processing such as heat treatment is needed, depending on the intended part properties or specific material characteristics [1]. Industrial applications for SLM are, for example, the production of tools and moulds, turbine parts for the aeronautic industry, and customized implants or other medical parts [1]. The fabrication costs of this process are basically dependent on the manufacturing time. For this reason, SLM has been used for single-part production. However, for several years, its popularity as an industrial manufacturing process is increasing [5].

Some basic approaches for welding SLM parts can be found in familiar investigations from previous years. For instance, an investigation on the electron beam welding of AlSi10Mg parts made through SLM showed a smaller heat affected zone (HAZ) compared to the joining of parts made by conventional casting, whereas the weld seam of the casted parts had a lower level of porosity [6]. For some light metals, a joining through friction welding is possible. When AlSi12 processed by SLM is used in a friction welding process, a decrease of the yield strength and an increase of the ductility were observed at once, in comparison to the joining of casted material [7]. The work of Casalino et al. [3] demonstrates the possibility of welding SLM parts by the usage of a laser-arc-hybrid process. In addition, a comparison with the welding of conventionally fabricated sheet material was made. The welds executed with the laser-leading configuration were nearly free of defects. The main advantage of a conventional arc welding technique is due to the processes capability of tolerating small gaps between the parts.

Laser beam welding is a promising technology for the joining of SLM parts, as the fine component microstructure remains nearly unchanged because of low heat input and high cooling rates. Another reason why laser beam welding is suitable in this application case is given by the fact, that SLM parts show a low level of porosity in general, which is known to affect the quality of a weld negatively [8]. 

Matilainen et al. [4] investigated the application of laser beam welding to join SLM parts made of 316 L stainless steel in comparison to the welding of cold rolled steel sheets. The results show that laser beam welding is feasible to join SLM parts with an acceptable weld quality. To realize a full penetration of the material, lower laser powers were needed. In the case of an insufficient welding depth, the SLM-processed material tended to cause a higher level of porosity. When it comes to high-energy input, the SLM-fabricated material showed a higher tendency to cracking, compared to the cold rolled samples. However, this shows the necessity of further investigations on the correlation between SLM process parameters and the characteristics of the weld seam, as well as the execution of comprehensive experiments to realize reproducible welds of high quality.

The aim of this work was laser beam welding of SLM tubes with a reproducible quality. This was a representative geometry for circumferential seams, as they occur, for example, in burners. For this purpose, destructive as well as non-destructive tests were carried out. The quality of laser-welded joints was assessed according to the requirements of DIN EN ISO 5817 [9] and DIN EN ISO 13919-1 [10]. The target was to achieve the criteria of evaluation group B.

## 3. Material and Methods

### 3.1. Materials

In the welding tests, SLM-manufactured tubes made ofnickel-based superalloys Inconel 625 (IN625) and Inconel 718 (IN718) were used. The tubes, with a length of 100 mm, were welded together. The wall thickness was 2.7 mm and the diameter of the tubes was 33 mm. The chemical composition according to the specifications of the materials is shown in Table 1.

Half of the tubes were subjected to a heat treatment before welding. No post heat treatment was applied after welding. For both materials, the heat treatments recommended by the manufacturer were made, which provided the best mechanical and high temperature properties. The heat treatment parameters for both materials are given in Table 2.

The gas mixture Atal (18% CO_2_ in argon) was used as process gas and pure argon for the root forming.

### 3.2. Experimental Procedure

**Figure 1:**
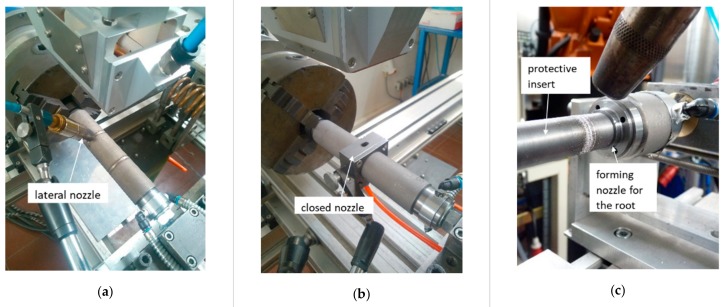
Experimental setup: (**a**) the variant with the lateral nozzle, (**b**) the variant with the closed nozzle, (**c**) the view of the forming nozzle for the root forming gas and protective insert.

The welding tests were carried out with laser beam welding technique without the addition of any filler material. The laser used was a 16 kW Yb:YAG disk laser and a laser optic YW32, with a scan tracker from Precitec as welding optics. The laser beam was transmitted by an optical fiber with a core diameter of 200 µm and focused to the diameter of 420 µm. The experimental setup is shown in Figure 1. The pipes were welded in a rotating manner. The position of the welding optics remained constant during the process. Two types of process gas nozzles were tested. In the first phase of the experiments, the process gas was fed through a lateral nozzle with a setting angle to the laser axis of 45 degrees. Furthermore, tests with a closed gas nozzle were carried out, which offered a gas cover in the full seam circumference. In addition, a forming nozzle was installed in the rotating device to ensure the supply of the root forming gas. A protective insert was also used to prevent damage of the inside of the tube due to the passage of unabsorbed laser power.

Preliminary tests were carried out to determine the welding parameters, which can be applied for the production of a series of welds with a reproducible quality for further evaluation. From previous research, it is known that the formation of an end crater in the overlap area is to be expected when closing a circular weld. For this reason, preliminary tests were also focused on avoiding this imperfection. All welds were butt-welded with a technical zero gap. The quality of the edge preparation was either SLM or mechanically cut edges. The laser power (P_l_) was varied in the range between 1600 W and 2400 W, and the welding speed (v_w_) was in the range from 1 m/min to 1.5 m/min. The focus position of the laser beam was on the tube surface. In addition, the methods known from laser beam welding were tested to see if modified parameters, the use of a laser scanner, and the variation in amplitude will reduce pore formation.

For evaluation, three samples per material combination were subjected to computed tomographic as well as metallographic examinations, as well as to residual stress measurements. Considering two different materials, heat treatment conditions and edge quality, a total of 24 samples and one calibration sample were tested. The overview of the samples is shown in Table 3.

### 3.3. Evaluation Tools

The evaluation of the welded joint was based on the standards DIN EN ISO 5817 [9] and DIN EN ISO 13919-1 [10]. In this way, both the material and the welding process were evaluated. These two standards describe the quality of a weld based on the type, size, and number of selected irregularities. For this reason, residual stress measurements, computed tomography, as well as metallographic investigations and hardness measurements were applied.

#### 3.3.1. Residual Stress Measurements

**Figure 2:**
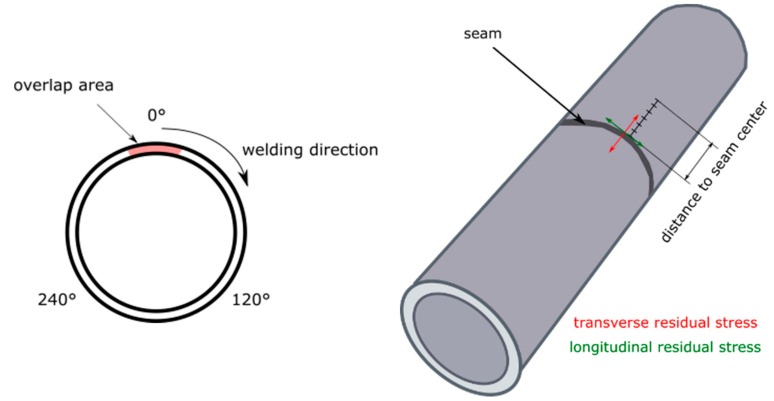
Measuring points of the residual stress.

The laser-welded samples were examined by means of radiographic residual stress measurements. First, the residual stress values were evaluated on the calibration sample in 30 measuring points. This quantity resulted from 5 measuring points × 2 measuring directions × 3 points in the seam circumference. The three measuring points were located in the seam circumference. The distance between the measuring points was 120 degrees. Therein, a measuring point was placed in the overlap area. After evaluation of the measurement results, the most critical point of the weld and the measuring direction with the highest residual stress values were decided. In the next step, all further residual stress measurements were made only at one point of the weld and in one measuring direction at five measuring points per weld sample. Figure 2 shows the measuring points of the transverse and longitudinal residual stresses in the overlap area 0° and with an offset of 120° and 240° for different distances to the seam.

#### 3.3.2. Computed Tomography

All 24 samples were subjected to a micro–computed tomography (µ-CT) test. For this purpose, the calibration sample was used to calibrate and adjust the computed tomography (CT) testing system. After successful adjustment, the internal imperfections of the welds were measured and quantified. The measurement results were evaluated in accordance with evaluation groups of DIN EN ISO 13919-1 [10]. For a material thickness of 2.7 mm, valuation groups were derived, which can be found in Table 4. To identify the related valuation group, the dimension of the pores was measured individually. The sum of the projection area of the pores was compared to the total area of the seam, A_total_, which was calculated as follows, where d is the diameter of the tubes and w the width of the seam:

A_total_ = π·d·w,

A_total_ = π·33 mm·2.6 mm = 269.5 mm².

The average weld width w could either be measured directly on welded samples with a linear measuring device, or planimetrically on metallographic cross sections.

#### 3.3.3. Metallographic Investigations and Hardness Measurements

**Figure 3:**
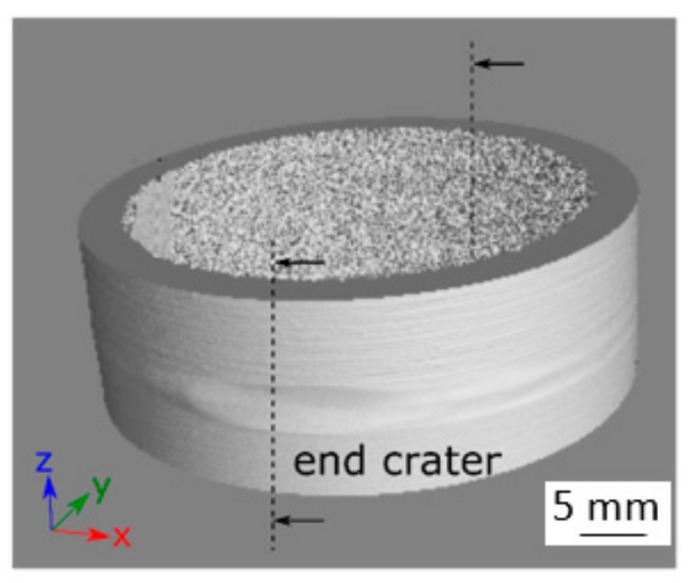
Circumferential weld in three-dimensional projection (3D), showing locations for the taking of cross sections.

After the non-destructive tests, the samples were post-processed by machine, metallographically prepared, and examined. For the metallographic investigations using light microscopy, a total of 48 cross sections were prepared and examined. The end crater and the opposite side were always considered for each weld. The locations of the cross sections are shown in Figure 3.

Hardness measurements HV1 with at least 10 measuring points for each weld were carried out. The hardness values were taken in the middle of the tube wall. The Vickers hardness test was carried out according to DIN EN ISO 6507-1 [11], and the hardness testing machine was calibrated according to DIN EN ISO 6507-3 [12]. The maximum deviation of the hardness measurements was in the range ±3%.

## 4. Results

### 4.1. Optical Evaluation of the Welding Results

For the evaluation of the welding results, the seams were assessed for their external quality. For this, undercuts, excessive root-side drop-through, spatter formation, and smoke deposition, among others, were used as quality characteristics. 

**Figure 4:**
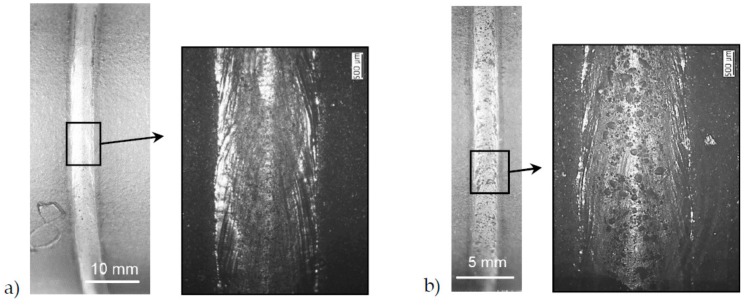
Welded samples of IN718 with different edge quality: (**a**) with machined edge, and (**b**) with SLM edge.

There were no differences in outer weld quality when comparing IN625 and IN718. Heat treatment conditions also resulted in no differences in weld bead formation. A significant difference in the quality of the seam surface was found when comparing the SLM edge and the mechanically cut edge. As Figure 4a shows, welded samples with a machined edge had a shiny surface. The samples with SLM edge showed an agglomeration of silicate-like inclusions on the top of the seam (Figure 4b). It was supposed that these inclusions were present on the top surface of the SLM edges and floated toward the surface after the weld edges melted. 

Despite the good weld quality in the seam circumference, the overlap area was the critical area of the weld. End crater cavities formed at the end of the process when closing the circular weld.

**Figure 5:**
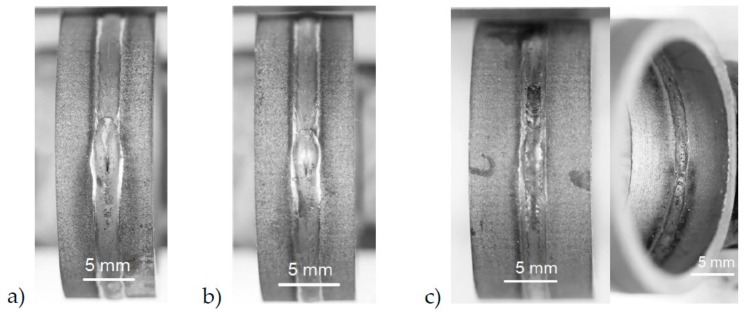
End crater outer appearance in overlap area at different process parameters: (**a**) P_l_ 1700 W, v_w_ 1.0 m/min, RP_l_ 200 ms; (**b**) P_l_ 1600 W, v_w_ 1.0 m/min, RP_l_ 200 ms; (**c**) P_l_ 1600 W, v_w_ 1.0 m/min, RP_l_ 200 ms, scanner frequency (f) 100 Hz, and amplitude (A) 1 mm.

A laser power ramp (RP_l_) before the process end with the ramp time in the range of 200 ms to 700 ms was tested to avoid abrupt shutdown of the laser and to prevent formation of the end crater. Such a simple method did not result in effective suppression of the end crater.However, it could be shown that the size of the end crater cavity depended on the laser power applied. In other words, the size of the end crater had a correlation to the volume of the molten metal in the end crater area. Figure 5 illustrates this observation. While an increased laser power of 1700 W leads to a pronounced end crater notch (Figure 5a), the end crater became significantly smaller at a lower laser power of 1600 W (Figure 5b).

This effect was due to an interaction between the molten pool volume, the melt flow dynamic, the viscosity, and the cooling rate of the melt. The end crater could not be completely avoided by adjusting the laser power. 

Laser beam oscillation was used in combination with the laser power ramp to provide more favorable movement of the melt for end crater suppression. The scan tracker of the optics was switched on 500 ms before the end of the process, corresponding to approximately 8 mm of the weld length at the welding speed of 1 m/min. As a result, beam oscillation transversally to the welding direction, having a scanning amplitude (A) of 1 mm and a frequency (f) of 100 Hz, caused a molten pool movement, so that the confluence of the melt in the center of the end crater improved and the notch formation was suppressed. An end crater region optimized in this way can be seen in Figure 5c. The approaches known from laser beam welding can therefore be applied to the welding of SLM components.

### 4.2. Residual Stress Measurements

**Figure 6:**
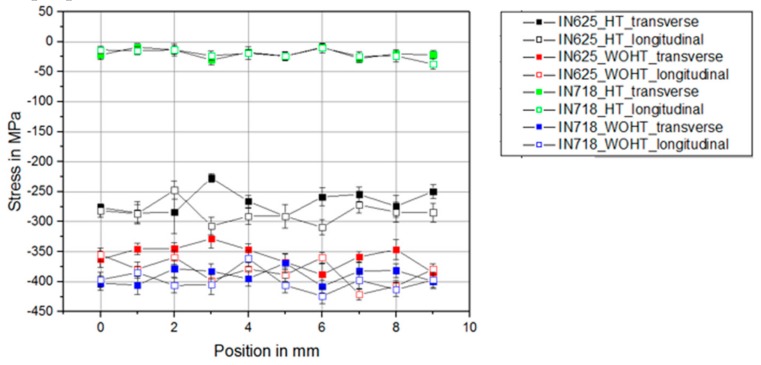
Residual stress profiles of the base SLM material.

As a first step, residual stress measurements were carried out on the base SLM materials IN625 and IN718, with and without heat treatment. The residual stress profiles were measured in both transversal and longitudinal directions to the tube axis. These values served as reference values for further investigations with the welded SLM samples (see Figure 6).

It could be determined that a heat treatment of the material IN718 reduced the residual compressive stress from 400 MPa to 30 MPa. With the IN625 material, the heat treatment had a significantly lower effect on the residual compressive stress. Thus, only a reduction from 360 MPa to 270 MPa could be observed here. The materials were heat-treated according to the parameters defined by the manufacturer. However, the parameters were applicable to cast or extruded material. Parameters for SLM-manufactured parts were not considered. Since the grain size of SLM-manufactured components was significantly smaller than that of cast components, this probably had an effect on the properties. 

There was no significant difference between the residual stress values for transversal and longitudinal directions for both materials.

**Figure 7:**
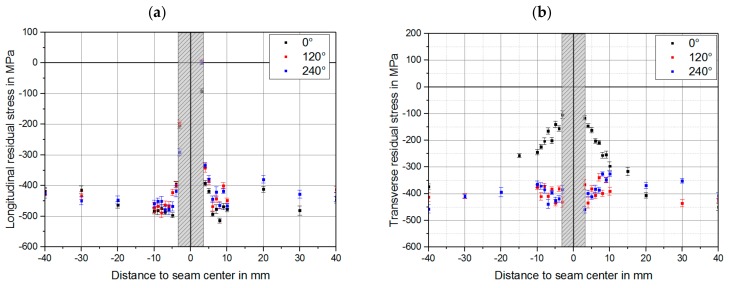
Comparison between residual stress values for the welded sample, IN625_WOHT_CT, in three segments in the weld circumference for (**a**) longitudinal direction and (**b**) transversal direction to the weld seam.

The next step was to identify the most critical area concerning the residual stress in the vicinity of the weld. The measurements were carried out on three areas of the weld circumference: in the overlap area (0°), and with an offset of 120° and 240°. A welded specimen, IN625_WOHT_CE, was used for the measurement. The comparison in Figure 7 shows that the highest increase in residual stress values in the vicinity of the weld bead compared to the base material was in the overlap area (0°), primarily the transverse residual stresses. For the specimen tested, the compressive residual stress values changed from 400 MPa to 100 MPa. The longitudinal residual stresses were nearly identical in the whole circumference. An evaluation of the residual stresses directly in the weld seam was not possible because of the high scattering of results. Therefore, the interpretable results can only be used from a distance of 3 mm to the weld seam center.

**Figure 8:**
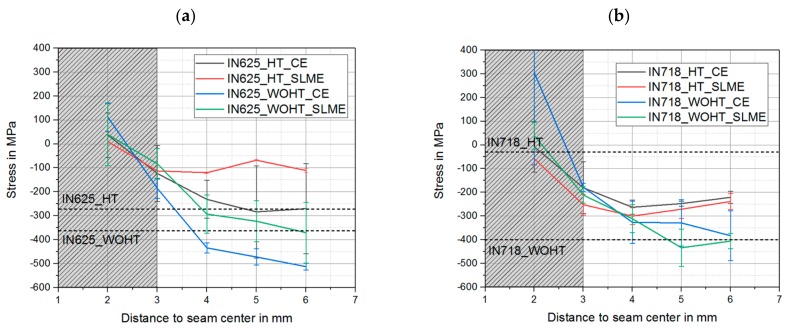
Residual stress profiles adjacent of the weld for both materials: (**a**) IN625 and (**b**) IN718.

The transverse residual stress measurements of the welded SLM-manufactured samples were carried out along the weld in the overlap area (0°), since the highest increase of the residual stress values compared to the conventional material was present and was thus defined as the most critical area. The residual stress profiles in the vicinity of the weld bead for both materials can be seen in Figure 8. The average transverse residual stress in the overlap area for each material and heat treatment condition are shown with dashed lines.

Comparing the results, it could be seen that for the material IN625, the compressive residual stress values reached 100 MPa at the distance of 3 mm from the weld seam center, corresponding to the results shown in Figure 8a. As the distance from the weld increased, the residual stress values tended to be closer to the base material values. Comparing these trends, the values of the heat-treated IN625 samples were higher, which again corresponds to the data from the measurements for the base material. In the case of IN718, the compressive residual stress values reached 200 MPa at the distance of 3 mm from the weld seam center (Figure 8b). As the distance from the weld increased, trends for the achieving of base material residual stresses were recognizable. It can clearly be seen that the values for the samples without heat treatment tended towards the direction of higher compressive residual stresses. For the samples with heat treatment, the trends were oriented upwards towards the lower level of compressive residual stresses, which corresponded to the results for the base material as to why a heat treatment should be preferred for laser beam welding of SLM-manufactured components.

A comparison of mechanically-cut edges and SLM edges showed no significant differences regarding residual stresses, especially for IN718. The differences of residual stress for IN625 resulted from a measurement uncertainty, as shown in the standard deviations in Figure 8.

### 4.3. Computed Tomography (CT)

**Figure 9:**
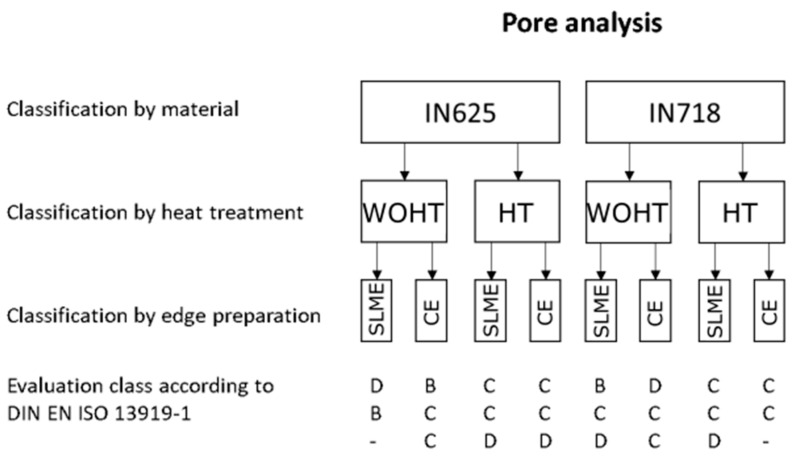
Results of quality evaluation in accordance with DIN EN ISO 13919-1 [10].

The CT measurement results were evaluated in accordance with DIN EN ISO 13919-1 [10], which specifies limit values for irregularities in the weld seam. The results of all samples are summarized in Figure 9.

In total, only 3 samples achieved category B, 13 samples were assigned to category C, 6 samples in category D, and 2 samples were not evaluable. The typical defects were pores and pores nests. In principle, the samples of IN625 without heat treatment showed fewer pores. No other trends or correlations between the material combination and pore formation could be derived. In general, the investigated laser welded SLM tubes tended to be classified in category C in terms of porosity.

**Figure 10:**
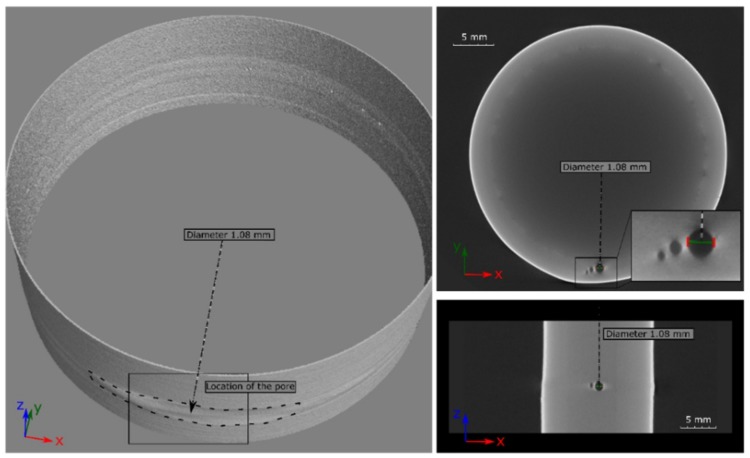
Projections of the computed tomography (CT) image for a laser beam-welded specimen IN625_WOHT_SLME classified to quality group C.

A 3D CT image of a laser beam-welded SLM-manufactured tube from IN625 without heat treatment and with an SLM edge is shown in Figure 10. It can be seen that the pores were concentrated in the overlap area. The diameter of the largest pore in this weld was 1.08 mm. Consequently, this welded sample can be classified in the evaluation group C, according to DIN EN ISO 13919-1 [10].

**Figure 11:**
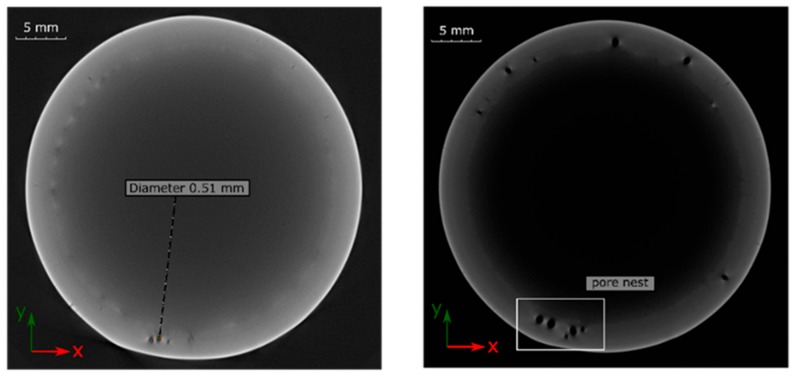
CT images of laser beam-welded specimens: (**a**) IN625_WOHT_SLME—group B and (**b**) IN718_WOHT_SLME—group D.

The CT image in Figure 11a demonstrates a sample that can be classified in the highest group B. Here, however, some subcritical pores were accumulated in the overlap area. Figure 11b shows a weld classified to lowest quality group D. Except for some isolated pores in the seam circumference, a pore nest can be also seen in the overlap area.

As the evaluation shows, the tendency towards excessive pore formation in the overlap area led to a classification of the weld quality into lower quality groups. This is a well-known phenomenon that occurs on the instability of the keyhole, especially at the closing of circumferential welds. 

There were several extensive studies available on the phenomenon of pore formation in laser beam welding [13]. The authors agree that the pores were caused predominantly through two mechanisms, which were keyhole instability [14] due to the pulsation of the metallic vapor flow, and the entrapping of ambient gas in the weld pool [15]. The nature of the pores also showed a mixed behavior between type one and two, indicating that the capillary was partially filled with vapor and partly with ambient gas [16]. The study [17] suggests that the porosity content correlates with the dimension of the molten pool. In other words, a larger molten pool leads to lower porosities. This is due to the longer time available for the degassing of trapped pores from the larger molten pool before it solidifies. In the work [18], it is shown that the dynamic of the melt, the stability of the keyhole, the weld solidification rate, and the pore escape rate are closely correlated with the welding parameters and material properties, and are the factors that determine pore formation. The authors of [19] reveal that for the welds on low carbon steel performed using a 2.4 kW CO_2_ laser power, the gas porosity can be eliminated by a weaving frequency of 22 Hz with 0.5 mm oscillating amplitude at 1 m/min welding speed. The research work [20] also shows that an effective suppression of porosity defects in the hybrid laser-arc welds on the high-strength low-carbon steel plates can be observed in the frequency range between 20 Hz to 40 Hz. Here, the recommended oscillating amplitude should be no more than 2 mm in order to achieve the keyhole mode of laser beam welding at the welding speed of 1 m/min. The effects of beam oscillating parameters on weld morphologies for austenitic stainless steel were investigated statistically in [21]. It was shown that there are threshold frequencies for different amplitudes to transform the welding from keyhole mode to unstable mode. According to this, with the amplitude of 1 mm and oscillating frequencies smaller than 100 Hz, the heat input is sufficient to maintain keyhole mode.

These considerations can also be applied to the phenomena of pore formation investigated in this work. Indeed, during the laser beam welding of SLM tubes with constant parameters (P_l_ = 1600 W, v_w_ = 1 m/min) the weld was fully penetrated, the keyhole kept open, and no obvious porosity occurred, aside from individual subcritical pores. In this case, the molten pool was large enough, which contributed to the pores spreading. The laser power ramp was applied with overlap when closing the weld, partially breaking the weld, and switching the keyhole to unstable mode. In this time, the pulsation of the vapor flow in the keyhole provoked the collision of molten metal, and then led to the formation of a liquid bridge and closed space in the keyhole. A pore was then produced.

On the basis of the known techniques from laser beam welding for circumferential welds of conventional components, an attempt was made to stabilize the keyhole and promote degassing of the pores by using a laser scanner. Although a certain improvement in pore avoidance in the overlap area could be achieved, complete pore removal could not be accomplished because the scanner parameters were not optimal. This was one of the reasons why the results of the quality assessment in Figure 9 are scattered.

### 4.4. Metallographic Investigations and Hardness Measurements

**Figure 12:**
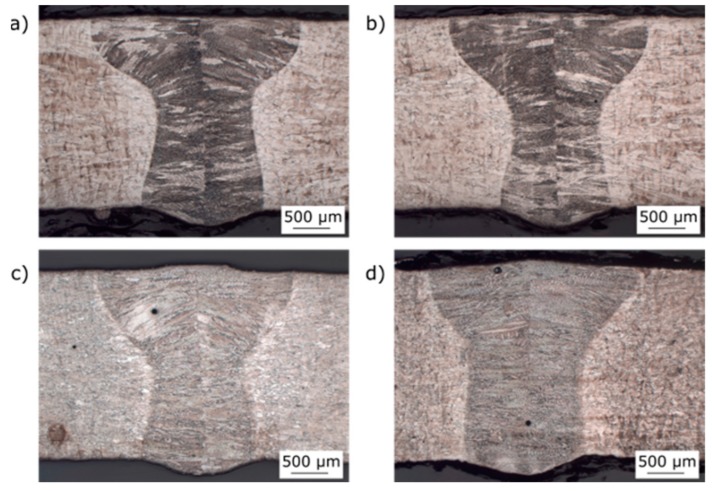
Cross sections of SLM manufactured tubes of IN625 and IN718: (**a**) IN625_HT_SLME, (**b**) IN625_WOHT_SLME, (**c**) IN718_HT_SLME, and (**d**) IN718_WOHT_SLME.

Within the metallographic process, 50 macro sections were made. One sample of each material used in this work with (HT) and without heat treatment (WOHT) are shown in Figure 12. 

These cross sections were taken from the seam circumference. Generally, the weldability of SLM-manufactured tubes with the laser beam welding process was illustrated successfully. The width of the seam on the upper side was approximately 2.7 mm, whereas it was only 2 mm on the root side. A narrow heat affected zone of 50 μm to 100 μm can also be identified.

**Figure 13:**
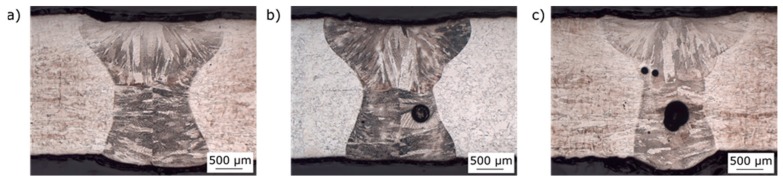
Cross sections of SLM-manufactured tubes of IN625 (overlap area): (**a**) IN625_WOHT_SLME, (**b**) IN625_HT_SLME, and (**c**) IN625_WOHT_SLME.

Again, cross sections were taken on the overlap area to show the most critical point of the welded samples with regard to the formation of pores, as shown in the results of the computed tomography analysis. It can be mentioned that an oscillating motion of the laser beam by a simultaneous reduction of the laser power in the overlap area effected a penetration of approximately 1.3 mm. Up to this penetration depth, the pores could be removed because of remelting. Nevertheless, the porosity in the overlap area could not be reproducibly suppressed due to instabilities of the keyhole in the end and variation of parameters. Cross-sections of SLM-manufactured tubes of IN625_SLME for each evaluation group according to DIN EN ISO 13919-1 [10] are shown in Figure 13. A correlation between the classification by material, edge preparation, heat treatment, and formation of pores could not be observed. As a positive aspect, it can be mentioned that no hot cracks can be seen in the welds produced, despite these tending to occur in the materials IN625 and IN718.

**Figure 14:**
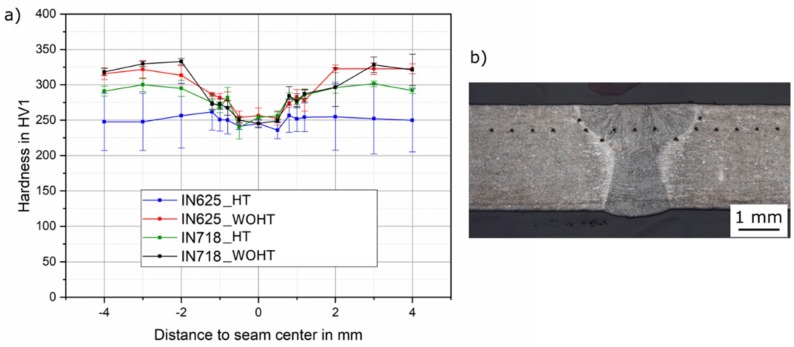
(**a**) Hardness measurements of SLM-manufactured tubes of IN625 and IN718, and (**b**) hardness of laser-welded IN718_HT_SLME as an example.

First, the hardness was measured above the plate thickness in three layers, each at a distance of 0.6 mm to the top and root side and in the middle of the tube thickness. The measured hardness values had no significant differences between the layer in the tube thickness. Therefore, the hardness was measured on the cap for the remaining samples. The mean hardness of the conventional material without heat treatment was approximately 310 HV1. Figure 14a shows a comparison of the measured hardness values for IN625 and IN718 with different heat treatment conditions. To evaluate the local hardness in the narrow heat affected zone, three hardness indentations were made. The average value of the hardness is shown in Figure 14b.

It can be observed that a previous heat treatment of the SLM manufactured tubes led to a reduction of the hardness in the base metal, especially for IN625. The average hardness in the heat-affected zone and in the weld metal were 290 and 260 HV1, respectively. A softening of approximately 20% in the weld metal and 7% in the HAZ was observed. A homogeneous course of the hardness over the entire weld zone is to be preferred. It is expected that the mechanical-technological properties of the weld joint were approximately on the same level, such as the base metal.

## 5. Conclusions

In summary, it was concluded that a welding of pipes made of Inconel could be shown successfully. However, the quality level (Group B) set as a target according to DIN EN ISO 13919-1 [10] could not be achieved. The results vary widely and tend to be assigned mainly to Group C. The individual results can be summarized as follows:

- An optical inspection of the weld seams revealed no differences between the materials IN625 and IN718. However, a clear variation was found in the weld preparation. Post-processed tubes showed a shiny surface compared to raw (SLM edge) tubes, which showed an agglomeration of silicate-like inclusions on the top of the seam. It was not excluded that these impurities remained in the weld metal and led to a deterioration of weld seam quality. For this reason, a mechanical processing of the edges is recommended.
- The residual stresses were considered, as they often are a problem for additive manufactured components. For this reason, the residual stress values of the welded tubes, which were either with heat treatment or without heat treatment, were compared with the residual stresses values of the base material. The results show that there were some differences between the values for welded tubes and base material, especially for IN718. A heat treatment is therefore preferable. The weld edge preparation had no significant effect on the residual stress values.
- The most difficult part in welding the tubes consisted of the formation of pores and pore nests. As can be seen from the CT images, the quality in the seam circumference can be classified as good. Pores were mainly formed in the end crater area, in the area where a keyhole instability was observed. The problem of the weld defects, such as pores and cracks in the overlap area of the circumferential welds, was already known. For this reason, the tests were carried out with already adapted parameters and a laser power ramp. However, pores and cracks in the overlap area could not be completely avoided by any method tested. For further tests, it is necessary to reduce the pores in the critical overlap area so that the weld seams can reach the highest quality, Group B, reliably and reproducibly. Hence, the interaction between scanning parameters, such as frequency and amplitude, and other welding parameters have still to be clarified. Such investigations must be carried out considering thermophysical properties of melt, such as surface tension and viscosity, to allow the laser beam oscillation technique to be transferred to SLM tubes made from nickel-based alloys to eliminate the formation of pores [22,23].
- Further tests need to be made to improve the welding quality in the end crater area by defocusing the laser [24].
- It can be mentioned positively that there were no hot cracks in the weld seams, despite the materials IN625 and IN718 being often susceptible to them.

## Figures and Tables

**Table 1:** Chemical composition of the materials in wt %. IN625: Inconel 625, IN718: Inconel 718.

Alloy	Ni	Cr	Mo	Fe	Nb	Mn	Si	Al	Ti	C	Co	Ta	B	Cu
**IN625**	bal.	21.5	9	2.5	3.6	0.2	0.2	0.2	0.2	0.05				
**IN718**	bal.	18.5	3	16.3	5.1	0.1	0.4	0.5	1	0.05	0.3	2.5	0.004	0.2

**Table 2:** Heat treatment parameters.

Material	Type of Heat Treatment	Atmosphere (mbar)	Temperature (°C)/Heating Rate (K/min)	Holding Time (min)	Cooling Conditions
**IN625**	solution annealing	<10^–4^ <10^–4^	1175/<25	60 60	40–60 K/min to 500 °C afterwards free cooling
**IN718**			982/<25		

**Table 3:** Material combinations for the welding. SLM: selective laser melting.

Material	Heat Treatment Conditions		Tube Edge Quality		Number of Samples for the Material Combination
	Heat Treatment (HT)	Without Heat Treatment (WOHT)	SLM Edges (SLME)	Mechanically Cut Edges (CE)	
IN625	+	-	+	-	3
IN625	+	-	-	+	3
IN625	-	+	+	-	3
IN625	-	+	-	+	3
IN718	+	-	+	-	3
IN718	+	-	-	+	3
IN718	-	+	+	-	3
IN718	-	+	-	+	3
Total number of samples					24

**Table 4:** Valuation groups in accordance to DIN EN ISO 13919-1 for material thickness 2.7 mm [10].

Irregularities	Remarks	Valuation Groups with Limits for Irregularities		
		Low D	Middle C	High B
Porosity and pores	Maximum size of a single pore in millimeters	1.35	1.1	0.83
	Maximum of the sum of the projection area of the irregularities in %	6	2	0.7
Pore nests and pore rows	Distance between individual pores in pore nests in millimeters	0.68	1.35	1.35
	Influenced weld seam length for pore nests in millimeters	5.4	2.7	2.7
